# Supplementary material for: Seizures Related to Influenza in Pediatric Patients: A Comparison with Seizures Associated with Other Respiratory Viral Infections
Source: J Clin Med. 2021 Jul 13;10(14):3088. doi: 10.3390/jcm10143088 (PMC8303985; doi:10.3390/jcm10143088)
Supplement: Supplementary file 1 [file jcm-10-03088-s001.zip › jcm-1280305-supplementary.pdf]

Table S1. Comparison between the influenza group and other respiratory viruses group excluding patients aged ≥5 years.

Factor	Influenza group (*n* = 97)	Other RVs group (*n* = 106)	*p*-value
Median age, years (range)	3 (0–17)	1 (0–4)	<0.001
Age group			
<5 years	75 (77.3)	106 (93.8)	<0.001
≥5 years	22 (22.7)	0 (0.0)	
Sex			
Male	61 (62.9)	69 (65.1)	0.743
Female	36 (37.1)	37 (34.9)	
Type of seizures			
Generalized tonic-clonic	79/93 (84.9)	91/105 (86.7)	0.942
Generalized tonic	10/93 (10.8)	10/105 (9.5)	
Others	4/93 (4.3)	4/105 (3.8)	
Seizure duration			
≤5 min	85/94 (90.4)	80/105 (76.2)	
≤15 min	9/94 (9.6)	15/105 (14.3)	0.011
≤30 min	0/94 (0.0)	8/105 (7.6)	
>30 min	0/94 (0.0)	2/105 (1.9)	
Number of seizures within 24 hours			
One	88 (90.7)	71 (67.0)	<0.001
Two	7 (7.2)	28 (26.4)	
Three or more	2 (2.1)	7 (6.6)	
Number of previous seizures with fever			
Null	42/96 (43.8)	45/106 (42.5)	
One	23/96 (24.0)	19/106 (17.9)	0.627
Two	10/96 (10.4)	15/106 (14.2)	
Three or more	21/96 (21.9)	27/106 (25.5)	
Time interval between fever and seizures			
<6 hours	30/93 (32.3)	38/102 (37.3)	
<12 hours	21/93 (22.6)	24/102 (23.5)	0.911
<24 hours	21/93 (22.6)	22/102 (21.6)	
<72 hours	20/93 (21.5)	17/102 (16.7)	
≥72 hours	1/93 (1.1)	1/102 (1.0)	
Abnormal EEG result	2/23 (8.7)	3/48 (6.3)	0.656
Abnormal brain MRI result	0/12 (0.0)	1/24 (4.2)	1.000
Family history of febrile seizures	24 (24.7)	37 (34.9)	0.115
Family history of epilepsy	1 (1.0)	0 (0.0)	0.478
Subsequent seizures with fever	16/35 (45.7)	30/68 (44.1)	0.877
Subsequent diagnosis of epilepsy	1/35 (2.9)	5/68 (7.4)	0.661

RV: respiratory virus; EEG: electroencephalography; MRI: magnetic resonance imaging.
